# Draft Genome Sequence of Glyphosate-Degrading Achromobacter insolitus Strain Kg 19 (VKM B-3295), Isolated from Agricultural Soil

**DOI:** 10.1128/MRA.00284-20

**Published:** 2020-04-23

**Authors:** Sergey V. Tarlachkov, Dmitry O. Epiktetov, Alexey V. Sviridov, Tatyana V. Shushkova, Inna T. Ermakova, Alexey A. Leontievsky

**Affiliations:** a G.K. Skryabin Institute of Biochemistry and Physiology of Microorganisms, Pushchino Scientific Center for Biological Research of the Russian Academy of Sciences, Federal Research Center, Pushchino, Russian Federation; b Branch of Shemyakin and Ovchinnikov Institute of Bioorganic Chemistry of the Russian Academy of Sciences, Pushchino, Russian Federation; Queens College

## Abstract

The genome of an Achromobacter insolitus strain isolated from an agricultural soil polluted with the herbicide glyphosate is reported. The genome size is 6.4 Mb, with an average G+C content of 65.2%. These genomic data could contribute to a better understanding of the biochemistry and regulatory mechanisms of the microbial degradation of glyphosate and aminomethylphosphonate.

## ANNOUNCEMENT

Achromobacter insolitus strain Kg 19 is a bacterium isolated from soil samples taken from fields in the Krasnodar region of Russia (45°03′10.8″N, 38°52′22.8″E) that were subject to consequent glyphosate (GP) treatments over prolonged periods. Isolation of the strain was carried out as follows: soil samples were incubated for 3 months at 28°C with periodic stirring and moistening with MS1 mineral medium (1) containing GP (18 mg kg^−1^ soil) as a component of the herbicide Roundup, inoculated into liquid MS1 medium with 10 g liter^−1^ glutamate and 0.5 g liter^−1^ GP, and incubated on a shaker with sequential passages twice per week (20 passages in total). The resulting microbial associations were inoculated onto LB agar plates; the cultures were isolated according to the morphological distinctiveness of the colonies and cell size, shape, and mobility.

*A. insolitus* Kg 19 cells, as a pure culture, were found to be Gram-negative mobile short rods approximately 1.4 to 2.5 × 0.6 μm with an optimal temperature of 28 to 30°C forming cream-colored glossy colonies. The strain was routinely maintained on agar plates with MS1 mineral medium supplemented with 10 g liter^−1^ glutamate and 0.5 g liter^−1^ GP as the sole sources of carbon and phosphorus, respectively. *A. insolitus* Kg 19 obviously possessed enzyme systems capable of breaking the carbon-phosphorus (C–P) bond in a range of organophosphonates (OPn), as its growth was observed while cultivated under the same conditions but with 0.3 g liter^−1^ methylphosphonic acid, 0.35 g liter^−1^ 2-aminoethylphosphonate (2-AEP), or 0.33 g liter^−1^ aminomethylphosphonate (AMP) (all from Sigma-Aldrich, USA) as the sole phosphorus source.

For genome sequencing, DNA isolation was performed using a FastDNA Spin kit and FastPrep-24 homogenizer (MP Biomedicals, USA). The short-read library was prepared with a HyperPlus kit (Kapa, USA) in accordance with the manufacturer’s recommendations. In short, preparation included the following steps: enzymatic DNA fragmentation, end repair and A tailing, ligation of specific adapters, and amplification of the obtained library (7 cycles). The library was sequenced on a NovaSeq 6000 platform (Illumina, USA) with a NovaSeq 6000 S2 reagent kit (200 cycles).

Default parameters were used for all software unless otherwise noted. The quality of the reads was checked with FastQC v0.11.8 (2). Adapter sequences and low-quality regions in raw reads were cut with Trimmomatic v0.39 (3) using the following options: ILLUMINACLIP:TruSeq3-PE-2.fa:2:30:10; SLIDINGWINDOW:4:15; and MINLEN:30. After trimming, the clean reads were assembled using SPAdes v3.14.0 (4) with the following options: --cov-cutoff, auto; and --careful. The assembly quality was assessed with QUAST v5.0.2 (5). Genome annotation was performed using the NCBI Prokaryotic Genome Annotation Pipeline (6).

A total of 4.3 × 10^7^ paired-end reads with an average length of 101 bp (4.4 Gb) were obtained from the sequencing. As a result, 4.2 × 10^7^ clean reads with an average length of 99 bp (4.1 Gb) were assembled into 43 scaffolds with 630-fold coverage. The scaffold *N*_50_ value is 628,760 bp, and the largest scaffold is 973,458 bp. The genome size is 6.4 Mb, with an average G+C content of 65.2%. A total of 5,917 protein-coding genes (481 of which encode hypothetical proteins), 55 tRNAs, 6 complete or partial rRNAs, and 4 noncoding RNAs (ncRNAs) were predicted.

The gene *PhnJ*, which encodes alpha-d-ribose 1-methylphosphonate 5-phosphate C–P lyase, a pivotal protein of OPn-degrading enzyme complex (7), was discovered in *A. insolitus* Kg 19 as a part of the complete C–P lyase operon. The presence of the gene *phnX* encoding phosphonatase (phosphonoacetaldehyde hydrolase), the terminal hydrolase of the 2-AEP utilization pathway, was demonstrated as well. This corresponds with the range of utilized OPn substrates and makes *A. insolitu*s Kg 19 a promising object of studies of OPn metabolism in bacteria and a possible candidate for the development of novel methods of bioremediation of soil and water polluted by GP or AMP.

### Data availability.

The raw reads have been deposited in the NCBI SRA under the accession no. SRR11236102, and the whole-genome shotgun project has been deposited in DDBJ/ENA/GenBank under the accession no. JAALEO000000000. The version described in this paper is the first version, JAALEO010000000.
